# Managing Chronic Pain in the Elderly: An Overview of the Recent Therapeutic Advancements

**DOI:** 10.7759/cureus.3293

**Published:** 2018-09-13

**Authors:** Asad Ali, Abdul Wahab Arif, Chandur Bhan, Deepak Kumar, Muhammad Bilal Malik, Zohaib Sayyed, Khawaja Hassan Akhtar, Malik Qistas Ahmad

**Affiliations:** 1 Medicine, CMH Lahore Medical College and Institute of Dentistry, Lahore, PAK; 2 Medicine, Shaikh Zayed Hospital, Lahore, PAK; 3 Internal Medicine, Chandka Medical College Hospital, Larkana, PAK; 4 Internal Medicine, Jinnah Postgraduate Medical Centre, Karachi, PAK; 5 Internal Medicine, Shifa College of Medicine, Islamabad, PAK; 6 Pediatrics, Shaikh Khalifa Bin Zayed Al-Nahyan Medical and Dental College, Bahawalpur, PAK; 7 Internal Medicine, King Edward Medical University/Mayo Hospital, Lahore, PAK; 8 Hematology/Oncology, University of Arizona Cancer Center, Tucson, USA

**Keywords:** chronic pain, opioids, non-steroidal anti-inflammatory drugs (nsaid), quality improvement, geriatrics, neuropathic pain, topical agents, chronic illnesses, targeted therapy, pain management

## Abstract

A majority of the elderly suffer from chronic pain that significantly alters their daily activities and imposes an enormous burden on health care. Multiple comorbidities and the risk of polypharmacy in the elderly make it a challenge to determine the appropriate drug, dosage, and maintenance of therapy. Opioids are the most commonly used agents for this purpose in the elderly. The aim of this article is to discuss both the current well-established therapies used for managing chronic pain in the elderly and also the emerging newer therapies.

## Introduction and background

The demographic transition in the recent decades has resulted in a significant increase in the aging population around the world. This, in turn, has resulted in an increased prevalence of various problems faced by the elderly. Chronic pain is one such common ailment reported in the elderly that also poses a significant economic burden on health care. Chronic pain is defined as the pain that lasts for three to six months or more than expected [1]. Similar to the figures in the general population, the prevalence of chronic pain in the elderly also varies widely from 15.2% in Malaysia [2] to 69.8% in Germany [3]. This is even higher (up to 83%) among the elderly in nursing homes [4]. Pain in the elderly may be underreported due to the misconception that it is a normal process of aging and also due to cognitive disturbances.

Pain mechanism

Various mechanisms are proposed for increased pain and pain sensitivity in the elderly. The first mechanism is due to physiological changes in the elderly like a decrease in neurotransmitters such as gamma-aminobutyric acid (GABA), serotonin, noradrenaline, and acetylcholine, decrease in the number of peripheral nociceptive neurons, increased pain thresholds, and reduced endogenous analgesic responses resulting in a paradoxical increase in the pain. Another reason is homeostenosis in aging, which is the loss of homeostatic reserve of various organ systems manifested as liver and renal function decline, decreased muscle mass and increased frailty leading to falls, decreased appetite, sleep disturbances, depression, delirium, agitation, and overall debility [5].

Causes of chronic pain in the elderly

Musculoskeletal disorders such as degenerative spine and arthritic conditions are the most common cause of chronic pain in the elderly. Other common causes of significance include neuropathic pain, ischemic pain, and pain due to cancer as well as its treatment [6]. Among elderly women, there is a high prevalence of vertebral compression fractures causing pain and discomfort.

Challenges and barriers

Chronic pain in the elderly is associated with an increased incidence of adverse outcomes, including functional impairment, falls, depression, and sleep disturbances. Pain management in older persons differs significantly from that in younger persons. Concomitant chronic illnesses make pain evaluation and treatment more difficult in the elderly. Also, older people respond differently to various therapies, usually with lesser efficacy and more severe adverse reactions, including additional risks of polypharmacy and addictions. In addition to this, the majority of elderly living in nursing homes have some degree of cognitive impairment, which has an impact on their ability to report pain resulting in inadequate pain assessment and management [7]. There is a lack of evidence-based guidelines for the treatment of chronic pain in the elderly as studies tend to focus more on younger adults. However, in the recent past, there has been an increase in the number of studies focused primarily on pain control in the elderly.

Pain assessment

Before outlining a treatment plan, a comprehensive assessment of the patient is essential for making a specific diagnosis and to ensure targeted therapy whenever possible. The functional status of the patient and the patient's ability to perform daily activities must be evaluated to determine the degree of independence, level of need for caregivers, as well as overall quality of life [6]. It is important to note that the majority of the elderly have multiple chronic illnesses that may affect an accurate assessment of pain as well as the efficacy of treatment [8]. For assessing pain intensity, the available data support methods like visual analogy scale (VAS), verbal descriptor scale, and numerical rating scale. The VAS is a reliable method used usually in clinical and research settings as it is simple, has minimal intrusiveness, and is ease to administer. Still, it should be used with caution in elderly patients as many of them report difficulty in completing the VAS, or give incomplete responses making it more difficult to give them scores [9]. For assessing the sensory, affective, evaluative, and miscellaneous components of pain, the McGill Pain Questionnaire used usually has proven evidence for validity, reliability, and discriminative abilities but not age-related components [10]. Of particular importance in the elderly are various tools used for pain assessment in cognitively impaired persons. Scales using pictures like the Wong-Baker FACES scale can be used for the elderly with limited cognitive ability. In elderly persons with advanced dementia, behavioral observation is needed to identify the presence of pain, for which tools like Pain Assessment in Advanced Dementia (PAINAD) and Checklist on Nonverbal Pain Indicators (CNPI) can be used [11].

Therapeutics

Various treatment options are available for chronic pain management in the elderly including either pharmacological or non-pharmacological measures or both combined. A comprehensive approach to dealing with common sequelae such as depression, isolation, and physical disability is considered effective. Non-pharmacological measures are considered particularly important in elderly patients as they have a lower frequency of adverse reactions compared with pharmacologic approaches and their benefit is usually enhanced when combined with drug strategies. Effective non-pharmacological approaches include physical therapy, cognitive behavioral therapy, and most importantly, patient and caregiver education interventions [12, 13], while pharmacological treatment modalities include non-opioid and opioid medications, pain modulating drugs, topical agents, and other newer therapies.

## Review

Treatment guidelines

The most appropriate treatment guidelines for chronic pain in the elderly are by the American Geriatrics Society (AGS), which were first formulated in 1998 and recently updated with newer pharmacological approaches in 2009 [1]. According to these guidelines, treatment is initiated by a drug with least toxicity preferably by the oral route, scheduled dosing with short-acting drugs in case of episodic pain, and consideration of rational polypharmacy with drugs of complementary mechanisms of action whenever needed.

Due to significant overlap between chronic geriatric pain and pain due to cancer, the World Health Organization (WHO) recommendations for pain management listed below [14] are also followed by a few clinicians. (1) giving drugs ‘‘by the clock’’ (2) by mouth and (3) following ‘‘analgesic ladder’’ which includes: a. Mild pain - the first choice is acetaminophen. b. Mild to moderate pain or pain uncontrolled with acetaminophen - NSAIDs. c. Pain refractory to NSAIDs - weaker opioid agonist. d. For pain refractory to the previous plan, or severe pain - pure opioid agonist. e. Adjuvant medication may be used for synergism with the previous medications.

Current Therapeutics

Acetaminophen: American Geriatrics Society (AGS) recommends acetaminophen as the first-line agent for mild to moderate chronic pain in the elderly [1] due to its favorable safety profile. The maximum daily dosage of acetaminophen allowed is 4 g with dose adjustments up to 50-70% required in patients with hepatic dysfunction. Hepatotoxicity is another major concern due to unintentional overdosage of acetaminophen, to avoid which proper patient counseling is required. One meta-analysis of seven randomized controlled trials showed superior efficacy of acetaminophen compared to placebo in reducing moderate pain but inferior to NSAIDs for both pain reduction and physical functioning [15]. Acetaminophen is an effective agent for relieving symptoms of osteoarthritis and low back pain [16]. However, it is less effective for chronic inflammatory pain (such as the pain associated with rheumatoid arthritis) than NSAIDs [17]. Also, NSAIDs have a better effect as short‐term (e.g., six weeks) treatment for osteoarthritis pain and low back pain.

Non-steroidal anti-inflammatory drugs (NSAIDs): Oral non-steroidal anti-inflammatory drugs (NSAIDs) like naproxen, ibuprofen, diclofenac, and celecoxib can be used for chronic pain in the elderly when acetaminophen fails to control the pain effectively. NSAIDs are particularly helpful in treating an inflammatory type of pain where acetaminophen has limited efficacy. The American Geriatric Society recommends great caution while using NSAID therapy in the elderly due to its established gastrointestinal, renal, and cardiovascular side effects and also recommends giving NSAIDs preferably for only short periods of time during episodic flares. A study on drug reactions leading to hospitalizations in the elderly showed NSAID related side effects as the cause for hospitalization in 23.5% of the elderly [18]. Gastrointestinal toxicity (gastric complications causing iron-deficiency anemia) risk due to NSAIDs is dose-dependent, increases with age, and also increases with concomitant use of cardioprotective doses of aspirin, which is common in elderly. Nonacetylated NSAIDs like salsalate were found to have limited but not proven evidence of lower gastrointestinal toxicity compared to aspirin [19]. Gastrointestinal (GI) side effects were also found to be reduced but not completely eliminated with selective cyclooxygenase-2 (COX-2) inhibitor NSAIDs like celecoxib. However, the risk of other toxicities is still high with COX-2 inhibitors and warrants caution for elderly use [20]. Some COX-2 selective inhibitors were withdrawn from the market because of a higher incidence of cardiovascular events, including rofecoxib and valdecoxib [21]. Protection against GI toxicity is achieved to a certain extent with concomitant administration of proton pump inhibitor, H2 blocker, or misoprostol and, if required, Helicobacter pylori eradication therapy. A meta-analysis showed a superior cardiovascular safety profile of naproxen compared with other selective or nonselective COX inhibitor NSAIDs [22]. The overall decision regarding the administration of NSAIDs for chronic pain in the elderly must be individualized based on comorbidities and other risk factors.

Topical NSAIDs: Topical NSAIDs therapy comprises an important alternative to oral NSAIDs in the elderly because of its rare systemic side effects and very few cutaneous effects like rash or pruritis at the site of application. Topical NSAIDs are often used for knee or hand osteoarthritis (OA) related pain. Evidence from a recent randomized controlled trial showed the comparable efficacy of topical diclofenac sodium with that of oral NSAIDs in the treatment of knee osteoarthritis with fewer adverse effects [23]. This is because of increased delivery of drug to adjacent synovium without much systemic absorption. In the United States, there are two approved topical NSAID formulations for OA: diclofenac sodium topical solution 1.5% in 45.5% dimethyl sulfoxide solution and diclofenac sodium 1% gel. Another category of drugs are rubefacients, which include salicylate-containing drugs like trolamine salicylate or methyl salicylate that act by counter-irritation, but they have a lesser effect on chronic pain control compared to topical NSAIDs and increased risk of salicylate toxicity [24].

Capsaicin: Capsaicin is an active alkaloid derivative from the capsicum family that acts by reduction of substance P and cutaneous nociceptive fibers. It can be used for both neuropathic pain and musculoskeletal pain but mostly as an adjuvant analgesic. The major disadvantage of this therapy is a burning sensation, which is particularly high in patients with existing scarred skin conditions like psoriasis. This can be overcome by application of low concentration capsaicin multiple times or high concentration once to desensitize the area of application. Previously only two strengths were available, 0.075% for neuropathic pain and 0.025% for arthritic pain, which are given up to four times daily. The Food and Drug Administration (FDA) approved high-concentration capsaicin 8% patch (Qutenza) for neuropathic pain, which can provide pain relief for up to 12 weeks after a single 60-minute application [25].

Topical lidocaine: Lidocaine is available in various cream formulations and as patches. The only FDA-approved use of 5% lidocaine is for postherpetic neuralgia for which it is applied 12 hours daily. With only mild skin irritation as a side effect, it can be used for various other neuropathic pain conditions.

Opioid analgesics: Opioid analgesics are considered for managing severe pain or failed response to other treatments in the elderly. Although the short-term efficacy of opioid analgesics in chronic pain in the elderly is established, [26] limited evidence is available regarding the long-term efficacy because of the discontinuation of opioid therapy due to various poorly tolerable side effects [27]. Opioid analgesics are classified into strong opioids like morphine, hydromorphone, oxycodone, fentanyl, and buprenorphine and weak opioids such as tapentadol and tramadol. Weaker opioids, tapentadol, and tramadol have a dual mechanism of action, as both mu-opioid agonists and norepinephrine reuptake inhibitors. Tramadol was approved by the FDA but caution is warranted in its use due to adverse effects like sedation, cognitive impairment, and interaction with other medications. Due to its strong serotonin reuptake inhibitory action, caution must also be taken particularly when prescribing tramadol to an elderly patient on selective serotonin reuptake inhibitors to avoid serotonin syndrome. Tapentadol was also approved for this use by the FDA in 2008. Unlike tramadol, tapentadol has only weak effects on serotonin reuptake and has no active metabolites, but tapentadol must be used with caution in the elderly because of the risk of causing severe hypotension, respiratory depression, and sedation. Randomized placebo and active-controlled studies done on elderly patients older than 75 years with chronic pain showed the similar efficacy of tapentadol ER and oxycodone controlled-release groups; also, more GI adverse effects were reported in oxycodone groups than tapentadol [28]. The use of strong opioids for managing pain in the elderly warrants caution because of the increased half-life of the active drug metabolites. Both short and long-acting agents have similar efficacy but a randomized crossover study showed that transdermal fentanyl was preferred by patients to sustained release morphine not only because it provided better pain relief but also because of the high incidence of constipation in the morphine group [29]. 

A prerequisite before initiation of opioid therapy is the evaluation of risk versus benefit in view of its adverse effects, and efforts to reduce risks are mandatory. Administration of opioids in the elderly is done on a trial basis to titrate the effective dose reaching the therapeutic goal with minimal adverse effects, starting with the lowest possible dose and gradual titration. Respiratory depression is one of the major concerns of opioid therapy, though tolerance to this effect develops rapidly. Respiratory depression is particularly common in patients who increase their doses rapidly, patients using drugs like methadone with variable pharmacokinetics, patients with concomitant use of drugs like benzodiazepines or barbiturates. Elderly patients in particular with hepatic and renal dysfunction are at increased risk due to resultant drug accumulation, which warrants regular monitoring of parameters like glomerular filtration and dose adjustments [30, 31]. With due course of time, tolerance develops to the majority of the side effects of opioids (except constipation due to gastric hypomotility) like respiratory depression, sedation, nausea, and vomiting. Until the development of tolerance, patients are managed with combined administration of antiemetics and usage of assistive devices. Complications of long-term usage of opioids include suppressed production of pituitary, gonadal, hypothalamic, and adrenal hormones manifesting as depression, fatigue, and decreased libido [1]. With prolonged therapy, opioid abuse is another major concern. Every patient should be assessed for risk factors related to the potential abuse with available tools like opioid Risk Tool (ORT) and the SOAPP-R (revised Screener and Opioid Assessment for Patients with Pain) [32]. Although clinicians must remain vigilant about the possibility of misuse or abuse of opioid agents in all patients, older age is generally associated with a relatively lower risk for opioid misuse and abuse [33].

Anticonvulsants: Antiepileptic drugs such as carbamazepine, gabapentin, and pregabalin are mainly used for neuropathic pain. In elderly patients with renal impairment, dose adjustment of gabapentin and pregabalin is required. Carbamazepine currently is the first line therapy for neuralgia. Gabapentin and pregabalin are recommended to be taken in short courses (two to four months) for certain types of neuropathic pain including diabetic neuropathy, central neuropathic pain after spinal cord injury, postherpetic neuralgia, and fibromyalgia. A meta-analysis study assessing 300 mg of pregabalin daily for neuropathic pain showed 50% reduction in pain [34]. Gabapentin dosage must be carefully titrated starting with 100-300 mg daily, up to a maximum dose of 3600 mg [35].

Serotonin-norepinephrine reuptake inhibitors (SNRIs): SNRIs such as venlafaxine and duloxetine can be used for neuropathic pain in the elderly. SNRIs are generally well tolerated but side effects include hyponatremia, giddiness, nausea, and abdominal pain. Venlafaxine is given at a daily dose of 37.5 mg, which can be increased up to 300 mg particularly in elderly patients with associated depression. But its use in the elderly is still under controversy, and results of a few studies actually limit its use due to hypertensive episodes. On the contrary, duloxetine has no cardiovascular effects and it showed a 50% reduction in diabetic neuropathic pain compared to placebos [36]. Dose titration must be done carefully as most of the side effects are dose-dependent.

Tricyclic antidepressants (TCAs): Tricyclic antidepressants such as amitriptyline, desipramine, and nortriptyline are effective in the treatment of postherpetic neuralgia and painful peripheral diabetic neuropathy, but tertiary tricyclics like amitriptyline is usually avoided in elderly patients in view of a higher incidence of side effects. Nortriptyline is usually given at a starting daily dose of 25 mg up to 200 mg if an associated depression is present. Adverse effects include both due to anticholinergic and noradrenergic effects. QTc prolongation is a major complication, so an electrocardiogram screening is a must before starting tricyclic therapy [37].

Benzodiazepines: Use of benzodiazepines for chronic pain in the elderly is limited by its adverse effects like tendency to falls and cognitive decline. It was found that 10%-20% of the falls in the elderly are due to the use of benzodiazepines [38]. Both long and short-acting benzodiazepines are included on the Beers list [39] despite the evidence of both increasing the risk of falls in the elderly. But considering the above facts, the use of benzodiazepines is usually limited in the elderly to conditions where spasm muscle, anxiety, and pain coexist.

Muscle relaxants: Baclofen, a GABA-B agonist is recommended as a second‐line agent for neuropathic pain, usually used in patients with spasticity due to central nervous system injury, demyelinating conditions, and other neuromuscular disorders. The initial dose has to be low, gradually increased to minimize the side effects like dizziness, somnolence, and gastrointestinal symptoms and slowly tapered before discontinuation to avoid delirium and seizures [40].

Other Agents

Calcitonin acting by an unknown mechanism is effective as a second-line agent for neuropathic pain and also shown to be effective in treating pain due to osteoporotic vertebral compression fractures and in patients with bone metastases [41, 42]. Usual side effects include hypersensitivity reactions and nausea. In addition, patients require regular monitoring of calcium and phosphate levels. Another group of agents also useful in pain due to cancer metastases are bisphosphonates particularly pamidronate and clodronate. These agents must be used with caution in elderly patients with renal failure, and also there is an occasional risk of esophagitis and hypocalcemia [43].

Newer Therapies

Cannabinoids: Medicinal use of cannabinoids is legal and are now available by prescription. Cannabinoids act on two classic receptors CB1r and CB2r. CB1 receptors are found in the central and peripheral nervous systems, bone, heart, liver, lung, vascular endothelium, and reproductive system while CB2 receptors are primarily found in the immune system, also at lower levels in the nervous system. CB1 receptors are the ones responsible for psychotomimetic adverse effects and addiction, because of which selective CB2r agonists are being preferred [44]. A buccal spray preparation Sativex is available from GW Pharmaceuticals Ltd, UK, which contains both psychoactive (tetrahydrocannabinol) and non-psychoactive (cannabidiol) extract from the cannabis plant. It is used for the treatment of neuropathic pain in multiple sclerosis patients. Other preparations available are synthetically derived nabilone and dronabinol, which are taken orally. Both these preparations are primarily used for chemotherapy-induced nausea and vomiting with only minimal analgesic action. A recent study on elderly patients greater than 65 years of age using cannabinoids for various conditions showed a marked reduction in pain and also better safety profile compared to opioids, but the safety of long-term therapy is not established. Common side effects include euphoria, anxiety, psychosis, sedation, dizziness, cognitive effects, tachycardia, palpitations, and postural hypotension. Due to narrow therapeutic index, elderly in particular are at higher risk of dysphoric effects of cannabinoids. In addition, the elderly must be warned against potential dangers during driving because of the effects of cannabinoids on reaction time and motor control [45, 46].

Micronized NSAIDs: Micronized NSAIDs are developed using nanotechnology to increase the surface area of drug and thus their absorption, providing comparable efficacy to conventional formulations at lower doses with minimized adverse effects. Previously FDA-approved two micronized NSAIDs, diclofenac (Zorvolex) and indomethacin (Tivorbex) for the treatment of mild-moderate pain in adults. FDA approved another drug meloxicam (Vivlodex™) for osteoarthritis following data from a phase 3 study of 402 patients with osteoarthritis pain in the hip or knee, the results of which showed that micronized meloxicam achieved efficacy at 33% lower doses than currently available meloxicam products [47].

Upcoming Novel Therapies

Target opioids: Several newer opioid agents with better safety profiles such as target opioid and mixed opioid agonists are currently under development. Activation of the mu-opioid receptor (MOR receptor) results in G-protein mediated analgesic effect of opioids; the receptor activation also results in recruitment of B-arrestin which is responsible for respiratory depression and various other side effects of opioids. TRV130 (Oliceridine) is a new μ-receptor G-protein pathway selective (μ-GPS) modulator with low β-arrestin recruitment and thus acceptable safety profile. Studies showed that TRV130 showed similar efficacy to that of morphine but with a wider therapeutic window and better safety profile [48]. Efficacy of TRV130 for chronic pain has not been studied until now and is also not yet approved by FDA even for acute pain. Most of the traditionally available opioids are MOP agonists; a mixed opioid agonist with a target combination of mu (MOP) and nociceptin/orphanin FQ peptide (NOP) receptors is currently being studied [49]. Two new MOP/NOP ligands SR16435 and BU08028 compared with morphine in mouse models demonstrated higher nociceptive potential with reduced development of tolerance to analgesia [50]. A preclinical study comparing BU08028 with MOP receptor agonists in nonhuman primates showed that BU08028 did not cause any cardiovascular side effects or respiratory depression even at 10- to 30-fold higher doses. Unlike with morphine, there is no development of physical dependence with BU08028. The mixed MOP/NOP agonist still needs further studies in humans, but data currently available is showing better efficacy and abuse free potential.

## Conclusions

Treating chronic pain in the elderly population still poses many challenges. A multidisciplinary approach using multicomponent strategies is needed as the most efficacious and safest therapeutic option for this population. With the introduction of targeted therapy and newer emerging modalities of pain control in the clinical practice, we hope managing chronic pain becomes easy in the near future. Prescribing physicians should start treating the geriatric population with the lowest possible, tolerable doses aimed at causing less adverse effects and improving the quality of life.
